# Provider-sponsored insurers, medical loss ratios, and quality ratings in ACA marketplaces

**DOI:** 10.1093/haschl/qxag246

**Published:** 2026-09-10

**Authors:** Lynn M Hua, Hyein Cho

**Affiliations:** Robinson College of Business, Georgia State University, Atlanta, GA 30303, United States; Cornerstone Research, Boston, MA 02116, United States

**Keywords:** medical loss ratios, vertical integration, ACA marketplaces, health insurance regulation, provider-sponsored insurers, integrated delivery systems, quality ratings

## Abstract

**Introduction:**

The Affordable Care Act's (ACA) medical loss ratio (MLR) regulation requires individual market insurers to spend at least 80% of premium revenue on medical care and quality improvement, but evidence is limited on whether MLRs correspond to measured quality and how this differs by insurer organizational structure.

**Methods:**

Using CMS MLR financial reports for ACA marketplace insurers (2015-2023) linked to Quality Rating System ratings (2020-2023), we compared provider-sponsored insurers owned by or operating as part of integrated delivery systems with traditional insurers on MLRs, spending components, and quality, using adjusted regressions.

**Results:**

Among 2751 issuer-state-years, 581 (21.1%) were provider-sponsored. Provider-sponsored insurers had MLRs 5.8 percentage points higher than traditional insurers (95% CI, 2.0 to 9.7). Provider-sponsored insurers had higher overall quality ratings (0.65 stars; 95% CI, 0.36 to 0.94), despite reporting $5.5 lower quality improvement expenses per member per month (95% CI, −8.9 to −2.2). A 10 percentage point higher MLR was associated with 0.15 higher stars (95% CI, 0.09 to 0.21). Prior-year, but not current-year, quality improvement expenses were associated with higher subsequent ratings (0.04 stars per $10 per member per month; 95% CI, 0.02 to 0.07).

**Conclusion:**

Organizational structure may shape MLR interpretation; direct quality measures may complement MLR regulation.

Key Points

- Provider-sponsored insurers had higher quality ratings despite lower reported quality improvement expenses, suggesting that standard medical loss ratio expense reporting may not fully capture quality related activities in integrated payer-provider models.
- Reported quality improvement expenses in the prior year, but not the current year, were associated with higher quality ratings, consistent with a lag between reported quality improvement activities and measured performance.
- As insurer-provider integration becomes more common, direct quality measures may help policymakers interpret spending-based medical loss ratio regulation.

## Introduction

The Affordable Care Act's (ACA) Medical Loss Ratio (MLR) regulation requires individual market insurers to spend at least 80% of premium revenue on medical care and quality improvement, rebating the shortfall to enrollees.^1^ In 2025, 5.1 million individual market enrollees received rebates averaging $233.^2^ The regulation uses a minimum MLR threshold to encourage insurers to direct more premium revenue toward medical care and quality improvement rather than administrative costs or profit. Whether a higher MLR also corresponds to higher quality care, however, is not obvious. Because the MLR measures medical claims and reported quality improvement expenses relative to premium revenue, a higher ratio could reflect more resources directed toward services and activities likely to improve care. Alternatively, it could reflect higher-cost or less-efficient care delivery that raises spending without commensurate gains in outcomes. Which interpretation holds and whether it differs across insurer organizational models remains largely untested.

These questions have become increasingly salient as insurers and health care providers integrate. Providers have consolidated rapidly into vertically integrated health systems, which vary widely in size, ownership type, and geographic scope; by 2018, more than half of US physicians and 72% of hospitals were affiliated with health systems.^3^ A subset of these integrated systems also operate health plans. In the ACA marketplaces, this integration is most visible in provider-sponsored insurers: insurance organizations offering plans that are owned by, affiliated with, or operated as part of integrated health systems. Hospital ownership of provider-sponsored health plans rose from 18.3% of hospitals in 2018 to 27.2% in 2023, with especially high prevalence among nonprofit and medical school-affiliated hospitals.^4^ This growth has intensified policy interest in whether integrated payer-provider models can improve quality and reduce fragmentation or instead raise new regulatory and market power concerns.

Provider-sponsored insurers may differ from traditional insurers in ways relevant to MLR regulation. Because they both finance and deliver care, they may have distinct opportunities to coordinate care and support measured quality. At the same time, integration may complicate MLR interpretation because insurance and care delivery are financially linked within the same organization.^5^ Thus, whether higher MLRs and reported quality improvement expenses correspond to higher measured quality may differ across organizational forms.

Prior work on integrated insurer-provider organizations has focused largely on Medicare Advantage, where integrated plans have been associated with differences in premiums, quality ratings, and outcomes.^6-9^ In ACA marketplaces, provider-sponsored health plans have been associated with higher quality ratings, including for clinical quality and enrollee experience.^10,11^ Separately, research documents insurer responses to MLR rules, including the reporting of quality improvement expenses.^12-14^ However, prior marketplace studies have not linked provider-sponsored insurer status to MLR financial reports, and examined whether MLRs and reported quality improvement expenses correspond to measured quality.

We examined MLRs, reported quality improvement expenses, and quality ratings among Affordable Care Act marketplace issuers from 2015 through 2023. We compared provider-sponsored and traditional insurers and assessed whether MLRs and reported quality improvement spending were associated with measured quality.

## Data and methods

This observational study examined Affordable Care Act marketplace issuers from 2015 through 2023, using issuer-state-year as the primary unit of analysis. We compared provider-sponsored issuers with traditional issuers in MLRs, quality improvement expenses, and quality ratings. This study used publicly available, aggregate issuer-level data and did not involve identifiable human participants. Institutional review board approval was not sought because, based on guidance from Georgia State University, this study was determined not to be human subjects’ research. This study followed the Strengthening the Reporting of Observational Studies in Epidemiology (STROBE) reporting guidelines.

### Data sources

We used Centers for Medicare & Medicaid Services (CMS) MLR financial reports for insurers offering qualified health plans in Affordable Care Act marketplaces from 2015 through 2023.^15^ Following CMS terminology, an ″issuer″ refers to the state-level entity within a health insurance company responsible for insurance offerings in that state. Because CMS medical loss ratio data are reported at this level, our unit of analysis is the issuer-state-year. Throughout the article, we use ″insurer″ when referring broadly to insurance companies and ″issuer″ when referring to CMS reporting units or units of analysis. These reports include issuer-state-year measures of medical claims, quality improvement expenses, earned premiums, enrollment, and MLRs. We linked these reports to CMS plan characteristics files to measure plan type and metal tier composition within each issuer-state-year.

Quality outcomes were obtained from the CMS Quality Rating System, which assigns marketplace plans star ratings on a 1- to 5-point scale.^16^ The Quality Rating System includes an overall quality rating and domain-specific ratings for clinical quality (preventive services, chronic disease management, acute care), member experience (patient satisfaction, plan responsiveness, coordination of care), and plan administration (appeals processing, network accuracy). Because marketplace quality ratings were available beginning in 2020, analyses involving quality ratings were restricted to 2020 through 2023.

We linked MLR reports and quality ratings using Health Insurance Oversight System identifiers, state, and year. Because MLR financial data are reported at the issuer-state-year level and cannot be matched to individual plans, we constructed issuer-state-year quality measures by averaging plan-level CMS star ratings across all rated marketplace plans offered by the same issuer in the same state and year. Additional details on sample construction and variable definitions are provided in the Supplement.

### Identifying provider-sponsored status

The primary exposure was provider-sponsored issuer status. We classified issuers as provider-sponsored if they were owned by, affiliated with, or operated as part of an integrated delivery system with hospitals or substantial physician employment. We identified them through a multistep process combining string matching of known integrated health systems (eg, Kaiser Permanente, Geisinger Health Plan, UPMC Health Plan, and HealthPartners), linkage to the Agency for Healthcare Research and Quality (AHRQ) Compendium of U.S. Health Systems,^17^ and manual verification using issuer and health system websites.

Because we use Compendium editions spanning our study period, systems that became integrated through mergers or acquisitions are captured as they are reflected in successive editions.^18^ Issuers not classified as provider-sponsored are referred to as traditional issuers.

### Medical loss ratio and quality measures

We used issuer-reported CMS MLR financial reports, which include MLRs and component fields such as earned premiums, medical claims incurred, and quality improvement expenses. MLRs are calculated as medical claims incurred plus quality improvement expenses divided by earned premiums, following Section 2718 of the Affordable Care Act.^1^ Medical claims include payments to providers for covered services, prescription drugs, and capitation arrangements. Quality improvement expenses include activities to improve health outcomes, implement health information technology, prevent hospital readmissions, improve patient safety, and implement wellness and health promotion programs. We analyzed MLRs and separately examined related financial measures: medical claims per member per month (PMPM), quality improvement expenses PMPM, total medical spending PMPM, and premiums PMPM. Total medical spending was defined as medical claims plus quality improvement expenses.

Quality outcomes were based on CMS Quality Rating System star ratings. The primary quality outcome was the overall quality rating, measured on a 1-to-5-star scale, with higher values indicating higher quality. Secondary quality outcomes were domain-specific ratings for clinical quality, member experience, and plan administration.

### Statistical analysis

We first compared characteristics of provider-sponsored and traditional issuer-state-year observations, including enrollment, plan type composition, metal tier composition, financial measures, and quality ratings. We then estimated linear regression models comparing provider-sponsored issuers with traditional issuers, adjusting for log member-years, plan type composition, metal tier composition, state fixed effects, and year fixed effects. Standard errors were clustered at the issuer level.

To evaluate whether MLRs were associated with measured quality, we estimated adjusted models of overall quality ratings as a function of the MLR, provider-sponsored status, and their interaction, with MLRs centered at 80% and scaled in 10 percentage point increments. We also examined whether current-year and prior-year quality improvement expenses were associated with overall quality ratings, with quality improvement expenses scaled per $10 PMPM.

In additional analyses, we estimated linear probability and logistic regression models for whether an issuer reported a MLR below 0.80, the rebate threshold. All statistical tests were two-sided, and *P* values less than 0.05 were considered statistically significant. Analyses were conducted using Stata, release 18.0 (StataCorp).

## Study results

### Sample characteristics

The analytic sample included 2751 issuer-state-year observations from 2015 through 2023, of which 581 (21.1%) were provider-sponsored, and 2170 (78.9%) were traditional. The quality-rating sample included 401 issuer-state-year observations from 2020 through 2023. Provider-sponsored and traditional issuers differed in several observable characteristics (Table 1). In unadjusted comparisons, provider-sponsored issuers had lower enrollment, a higher share of HMO plans, higher MLRs, and overall quality ratings than traditional issuers.

**Table 1.** Characteristics of provider-sponsored and traditional ACA marketplace issuers, 2015-2023.

Panel A: full sample, 2015-2023			
Characteristic	Traditional issuers (*N* = 2170)	Provider-sponsored issuers (*N* = 581)	*P* Value
Medical Loss Ratio, %	91.3 (26.0)	93.9 (22.6)	0.025
Medical Claims, $ PMPM	1320 (1304)	1322 (1102)	0.972
Quality Improvement, $ PMPM	15 (19)	12 (14)	0.001
Total Medical Spending, $ PMPM	1335 (1314)	1334 (1110)	0.991
Premium, $ PMPM	1446 (1248)	1430 (943)	0.777
Enrollment, Member-Years	52 657 (105 129)	35 736 (92 868)	<0.001
HMO Plans, %	41.6 (47.6)	68.4 (44.0)	<0.001
Silver Tier Enrollment, %	59.4 (19.6)	58.7 (17.9)	0.454

Panel B: quality subsample, 2020-2023			
Characteristic	Traditional issuers (*N* = 288)	Provider-sponsored issuers (*N* = 113)	*P* Value
Overall Quality, Stars	3.01 (0.73)	3.68 (0.74)	<0.001
Clinical Quality, Stars	2.80 (0.80)	3.38 (0.85)	<0.001
Member Experience, Stars	3.09 (0.85)	3.34 (0.68)	0.005
Plan Administration, Stars	3.32 (0.64)	3.82 (0.88)	<0.001

Data are presented as mean (SD) unless otherwise indicated. PMPM indicates per member per month. *P* values are from unadjusted two-sample t-tests comparing provider-sponsored to traditional issuers. Quality ratings are issuer-state-year means of CMS-assigned plan-level star ratings, unweighted across plans. Data sources: CMS medical loss ratio financial reports, marketplace plan files, and Quality Rating System data.

### Medical loss ratios, components, and quality

After adjustment for enrollment, plan type, metal tier composition, and state and year fixed effects, provider-sponsored issuers had MLRs 5.8 percentage points higher than traditional issuers (95% CI, 2.0 to 9.7; Figure 1). Among the financial measures, reported quality improvement expenses were $5.5 PMPM lower among provider-sponsored issuers (95% CI, −8.9 to −2.2), while differences in medical claims, total medical spending, and premiums were not statistically significant. On quality, provider-sponsored issuers had higher overall ratings (0.65 stars; 95% CI, 0.36 to 0.94), with higher ratings for clinical quality (0.60; 95% CI, 0.27 to 0.93) and member experience (0.39; 95% CI, 0.14 to 0.64) and no significant difference in plan administration (0.19; 95% CI, −0.15 to 0.54).

**Figure 1.**
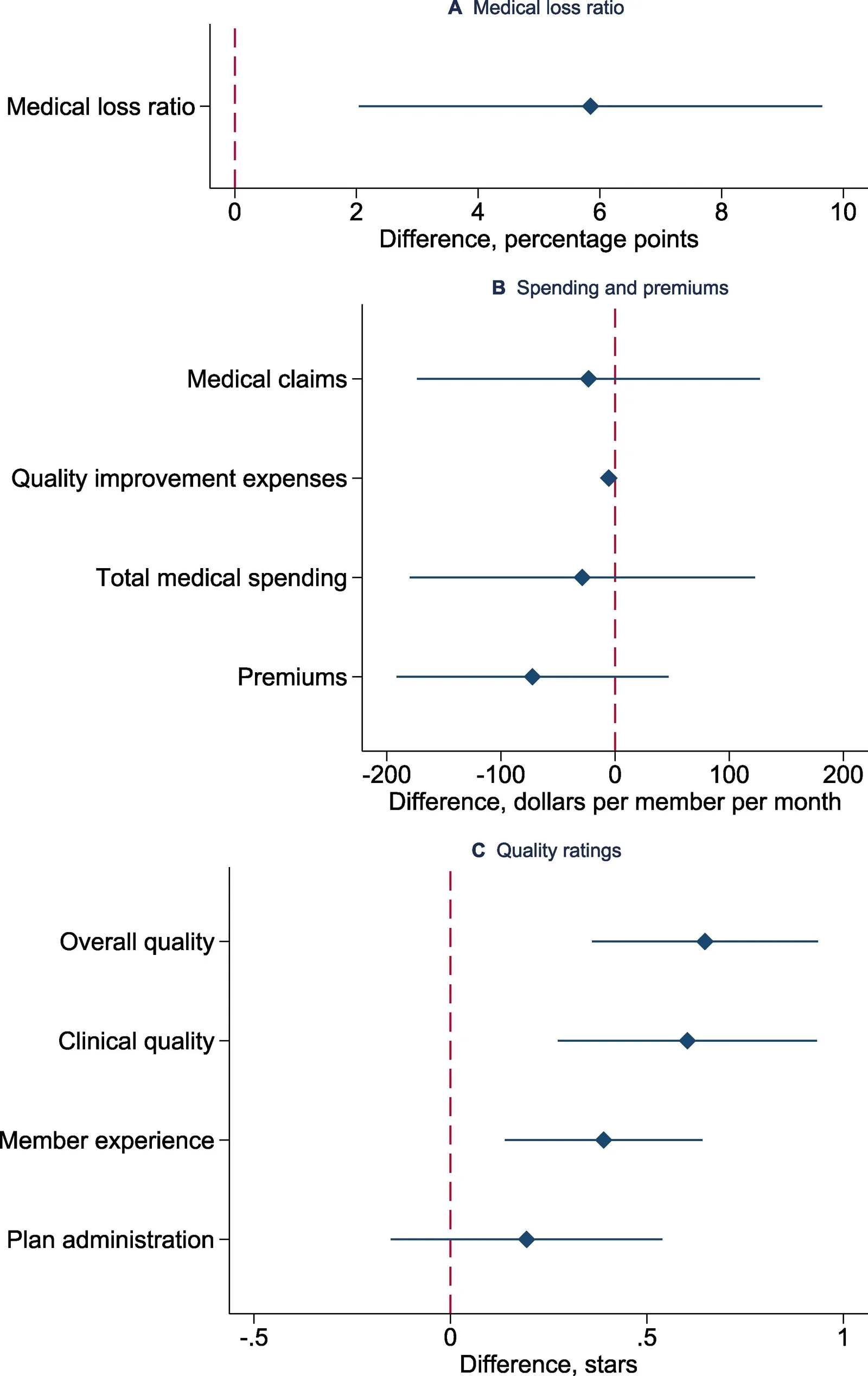
Adjusted associations between provider-sponsored issuer status and medical loss ratios, financial measures, and quality ratings. Notes: Each point represents the adjusted difference between provider-sponsored and traditional issuers, with 95% CIs, from models adjusting for plan type and metal tier shares, log member-years, and state and year fixed effects. Standard errors clustered by issuer. Panel A reports the medical loss ratio in percentage points, Panel B reports financial measures in dollars per member per month, and Panel C reports quality ratings in stars. Panels A and B are based on 2751 issuer-state-years from 2015 through 2023; Panel C is based on 401 issuer-state-years with quality ratings from 2020 through 2023. The dashed line marks a difference of zero.

Across multiple dimensions—including plan enrollment, delivery-system size, academic affiliation, Medicare Advantage participation, and geographic scope—associations between provider-sponsored status and both MLRs and quality ratings were broadly consistent rather than concentrated in a particular form of integration (Figure S1).

### Medical loss ratios, quality improvement expenses, and quality

Higher MLRs were associated with higher quality ratings: a 10 percentage point higher MLR was associated with 0.15 higher overall stars (95% CI, 0.09 to 0.21; Figure 2). The association did not differ detectably between provider-sponsored and traditional issuers (*P* = 0.25 for the interaction; Table S1). Current-year quality improvement expenses were not significantly associated with quality ratings, while prior-year quality improvement expenses were positively associated with subsequent quality ratings (0.04 higher stars per $10 PMPM, 95% CI, 0.02 to 0.07; Figure 3).

**Figure 2.**
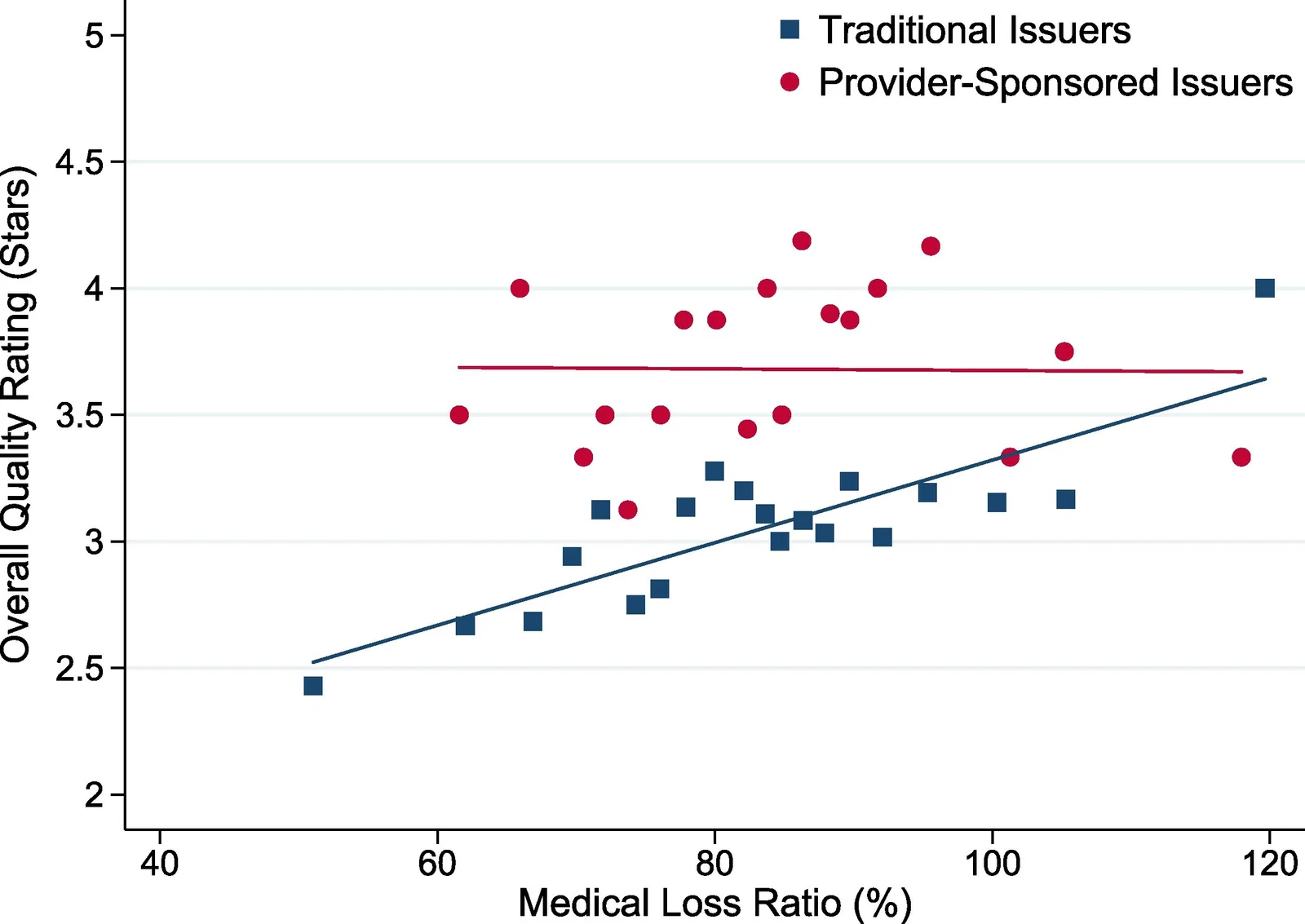
Association between overall quality rating and medical loss ratio, ACA marketplace issuers, 2020-2023. Notes: This figure shows overall CMS Quality Rating System ratings and medical loss ratios among marketplace issuer-state-years from 2020 through 2023, by issuer type. Points represent issuer-state-year binned means; lines show unadjusted linear fits estimated separately for provider-sponsored and traditional issuers, as indicated by the marker shapes and legend within the figure.

**Figure 3.**
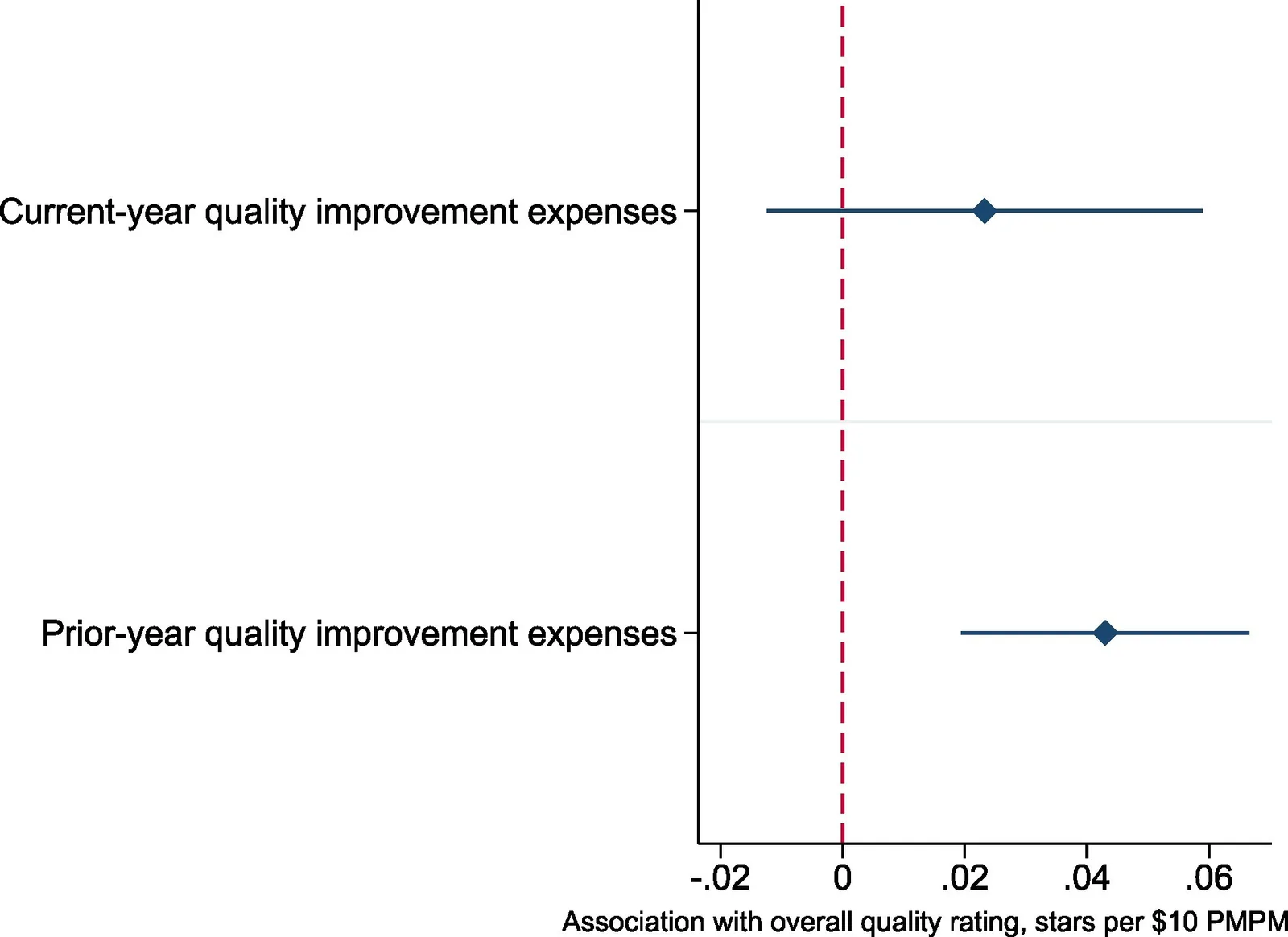
Current-year and prior-year quality improvement expenses and overall quality ratings. Notes: Each point represents the adjusted association between quality improvement expenses and overall CMS Quality System Rating, with 95% CIs. Quality improvement expenses are scaled per $10 per member per month. Estimates are from separate models for current-year quality improvement expenses (N = 401) and prior-year quality improvement expenses (N = 397). Models adjust for provider-sponsored issuer status, plan type shares, metal tier shares, log member-years, state fixed effects, and year fixed effects. Standard errors clustered by issuer. The dashed line marks an association of zero. PMPM indicates per member per month.

### Sensitivity analyses

The MLR association was similar across sensitivity analyses, including restricting the sample to 2020 through 2023, excluding 2020, and restricting the sample to larger issuers (Figure S2). Because the ACA marketplace experienced issuer entry and exit over the study period, these analyses assessed whether the main findings are sensitive to the study years or to the inclusion of smaller issuers. Provider-sponsored issuers were less likely than traditional issuers to report a MLR below 0.80, the ACA rebate threshold, a result similar in linear probability and logistic regression models (Figure S3).

## Discussion

In this observational study of Affordable Care Act marketplace insurers from 2015 through 2023, provider-sponsored insurers had higher MLRs and higher overall quality ratings than traditional insurers, despite reporting lower quality improvement expenses. Higher MLRs were positively associated with overall quality ratings, and prior-year quality improvement expenses were positively associated with subsequent quality ratings. These findings are consistent with prior marketplace evidence that provider-sponsored plans have higher quality ratings,^10,11^ and extend that work by linking provider-sponsored insurer status to MLR financial reports and by examining whether MLRs and reported quality improvement expenses are associated with measured quality.

These findings suggest that integrated organizational structures may complicate how reported quality improvement expenses are interpreted. Provider-sponsored insurers operate within delivery systems that may already maintain care management infrastructure, electronic health records, clinical protocols, and other quality related capabilities as part of care delivery. When these activities are embedded in clinical operations, they may not be reported as standalone quality improvement expenses in the same way as activities purchased or organized separately by traditional insurers. Thus, lower reported quality improvement expenses among provider-sponsored insurers may reflect differences in how quality related activities are organized or classified, rather than a lower commitment to quality.

The association between prior-year quality improvement expenses and subsequent quality ratings also suggests that timing may matter. Current-year reported quality improvement expenses were not significantly associated with overall quality ratings, whereas, prior-year expenses were positively associated with subsequent ratings. This pattern is consistent with a lag between reported quality improvement activities and measured performance, although the observational design cannot establish that reported quality improvement expenses caused higher ratings. More broadly, the positive association between MLRs and quality ratings suggests that MLRs may contain information about measured quality, but that relationship should be interpreted alongside direct quality measures.

Patterns were broadly similar across observable features of provider-sponsored insurers, including plan enrollment, delivery-system size, academic affiliation, Medicare Advantage participation, and geographic scope (Figure S1). These descriptive patterns suggest that the main findings were not obviously driven by a single observable subtype of provider-sponsored insurer. However, observable classifications may not capture important variation in how insurers and providers are integrated, such as sharing electronic medical records or aligning physician financial incentives. Future work linking insurer financial reports to more detailed measures of delivery system organization, plan design, provider networks, and enrollee outcomes would help distinguish these mechanisms.

Our findings complement research on integrated insurer-provider organizations in Medicare Advantage^6-9^ by examining ACA marketplace insurers, which serve a different population and operate under different regulatory rules. Recent Medicare Advantage evidence suggests that hospital-owned plans have higher MLRs than non-integrated plans,^6^ while prior marketplace studies have documented higher quality ratings among provider-sponsored plans.^10,11^ This study connects these strands by examining MLRs, reported quality improvement expenses, and quality ratings in the ACA marketplace setting.

These results have several policy implications. MLRs remain important indicators of the share of premium revenue directed toward medical care and quality improvement rather than administrative costs or profits. However, the same MLR may not be interpreted identically across organizational forms. When insurers and providers are financially integrated, reported medical spending may reflect multiple organizational, clinical, and reporting pathways. As integrated payer-provider models become more common, policymakers might consider how Quality Rating System performance can inform interpretation of MLR reports. Linking financial oversight with direct quality measures could help regulators assess whether higher reported medical spending corresponds to better measured care, rather than higher costs alone. Continued monitoring of integrated models is warranted to assess both their potential quality benefits and broader concerns about market power.^19^

### Limitations

This study has several limitations. Our observational design cannot establish causal effects and cannot rule out selection into provider-sponsored status. Provider-sponsored insurers may differ from traditional insurers in unobserved ways, including geographic concentration, market structure, provider networks, local delivery system characteristics, management practices, and enrollee composition. These differences could contribute to the observed associations between provider-sponsored status, MLRs, and quality ratings. We therefore interpret the estimates as associations rather than causal effects of integration.

Furthermore, provider-sponsored status represents a heterogeneous set of insurer-provider relationships. Due to data limitations, our classification cannot measure the depth or operational intensity of integration, including shared electronic health records, care management infrastructure, physician incentives, or financial alignment. We therefore cannot determine whether more tightly integrated systems account for the observed differences.

We observed reported expense categories rather than underlying activities or processes that generated them. Lower quality improvement expenses among provider-sponsored insurers may reflect differences in how quality related activities are classified or embedded within care delivery, rather than lower investment in quality. MLR data are reported at the issuer-state-year level and cannot be linked to individual plans, providers, or enrollees. We rely on the CMS Quality Rating System data, which were available starting in 2020, which shortened the quality analysis sample and constrained our ability to estimate multi-year lagged effects. QRS ratings may not capture all dimensions of quality, including access, appropriateness of care, long-term outcomes, or enrollee health.

## Conclusion

In this observational study of Affordable Care Act marketplace insurers, provider-sponsored insurers had higher MLRs and higher overall quality ratings than traditional insurers despite lower reported quality improvement expenses. These findings suggest that insurer organizational structure may shape the interpretation of MLRs in ACA marketplaces. As insurer-provider integration becomes more common, direct quality measures may be important complements to MLR regulation.

## Supplementary Material

qxag246_Supplementary_Data
