# Supplementary material for: Provider-sponsored insurers, medical loss ratios, and quality ratings in ACA marketplaces
Source: Health Aff Sch. 2026 Sep 10;4(10):qxag246. doi: 10.1093/haschl/qxag246 (PMC13637040; doi:10.1093/haschl/qxag246)
Supplement: Supplementary file 1 [file qxag246_supplementary_data.zip › qxag246_Supplementary_Data.pdf]

Provider-Sponsored Insurers, Medical Loss Ratios, and Quality Ratings in ACA Marketplaces

Supplementary Materials

Supplementary Methods

Supplement Figure 1. Association Between Provider-Sponsored Issuer Status and Medical Loss Ratios and Quality Ratings, by Observable Features of Provider-Sponsored Issuers

Supplement Table 1. Association Between Medical Loss Ratios and Overall Quality Ratings, Overall and by Issuer Type

Supplement Figure 2. Association Between Provider-Sponsored Issuer Status and Medical Loss Ratios and Quality Improvement Expenses Across Robustness Samples

Supplement Figure 3. Association Between Provider-Sponsored Issuer Status and Reporting a Medical Loss Ratio below 0.80

Supplementary Methods

Data Linkage

We constructed an issuer-state-year panel by linking CMS medical loss ratio financial reports to marketplace plan characteristics and Quality Rating System (QRS) ratings. Medical loss ratio reports are filed at the issuer-state-year level; marketplace plan characteristics and QRS star ratings are reported at the plan level. QRS star ratings were obtained from the CMS QRS Public Use Files and restricted to 2020-2023 due to data availability.

Plan-level data were aggregated to the issuer-state-year level before linkage. Plan characteristics were summarized as issuer-state-year shares (plan type and metal tier), and QRS star ratings were aggregated as the unweighted mean across an issuer's rated marketplace plans. These were merged to medical loss ratio reports using HIOS issuer identifiers, state, and year. Because medical loss ratio reports cannot be attributed to individual plans, all analyses were conducted at the issuer-state-year level.

Provider-Sponsored Issuer Classification

Provider-sponsored issuer status was assigned using a multistep classification process. First, issuer names and Health Insurance Oversight System identifiers were reviewed to identify well-known integrated delivery systems that operate health plans, including Kaiser Permanente, Geisinger Health Plan, UPMC Health Plan, and HealthPartners. Second, issuers were linked to the Agency for Healthcare Research and Quality (AHRQ) Compendium of U.S. Health Systems to identify relationships with hospital systems and physician-employing health systems. The Compendium is released as periodic editions, each of which derives the health-system landscape as of that release, incorporating systems that have grown through mergers and acquisitions. Third, issuer and health system websites were manually reviewed.

Issuers were classified as provider-sponsored if they were owned by, affiliated with, or operated as part of an integrated delivery system with hospitals or substantial physician employment. This classification was intended to capture integration between insurance and care delivery. It was not intended to capture narrower provider contracting arrangements, value-based payment contracts, narrow networks, or other relationships between traditional issuers and providers.

Variable Construction

Medical loss ratios were multiplied by 100 and analyzed in percentage points. Medical claims, quality improvement expenses, total medical spending, and premiums were analyzed per member per month. Total medical spending was defined as medical claims plus quality improvement expenses.

For analyses of the ACA rebate threshold, an indicator equal to 1 was constructed if an issuer-state-year reported a medical loss ratio below 0.80. In linear probability models, this outcome was multiplied by 100 so that coefficients could be interpreted as percentage point changes.

Logistic regression analyses report average marginal effects.

Statistical Analyses

The main adjusted models estimated the following specification:

$$Y_{ist} = \beta_0 + \beta_1 \cdot ProviderSponsored_{ist} + X_{ist}\gamma + \mu_s + \lambda_t + \varepsilon_{ist}$$

where Y_{ist} is the outcome for issuer *i* in state *s* in year *t*. $ProviderSponsored_{ist}$ is an indicator for provider-sponsored issuer status, X_{ist} includes plan type mix, metal tier distribution, and log member-years, μ_s represents state fixed effects, and λ_t represents year fixed effects. Standard errors were clustered at the issuer level.

For medical loss ratio, spending, premium, and quality outcomes, models were estimated using linear regression. Robustness analyses re-estimated the main models restricting to 2020–2023, excluding 2020, and restricting to issuers with at least 10,000 member-years.

Heterogeneity analyses replaced the provider-sponsored indicator with a categorical variable distinguishing traditional issuer from provider-sponsored subgroups defined along each dimension (**Supplement Figure 1**). The association between medical loss ratios and quality was estimated by regressing overall quality ratings on the medical loss ratio, provider-sponsored status, and their interaction (**Supplement Table 1**).

Supplement Figure 1. Association Between Provider-Sponsored Issuer Status and Medical Loss Ratios and Quality Ratings, by Observable Features of Provider-Sponsored Issuers

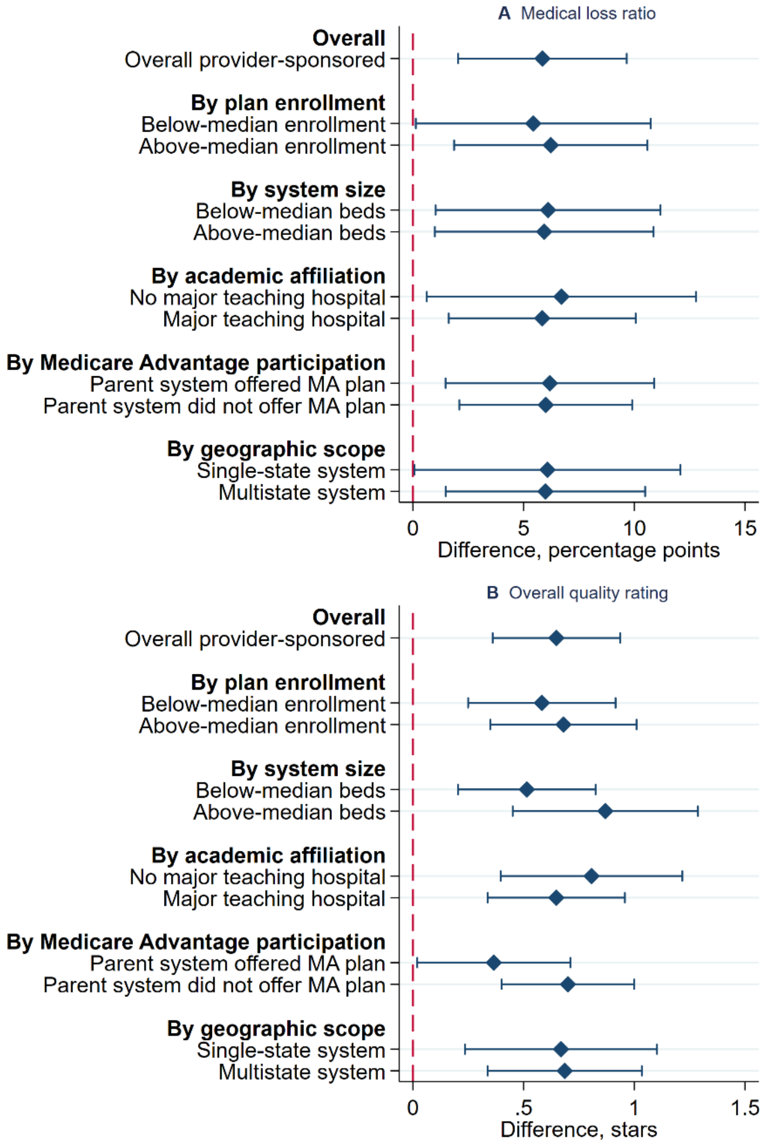

Notes: Each point represents the adjusted difference between a subgroup of provider-sponsored issuers and traditional issuers, with 95% CIs, estimated from separate linear regressions. Traditional issuers are the reference group. Panel A reports medical loss ratios in percentage points; Panel B reports overall quality ratings in stars. Within each panel, provider-sponsored issuers are disaggregated along five dimensions: plan enrollment, delivery-system size (hospital beds), academic affiliation (whether the system includes a major teaching hospital), Medicare Advantage participation (whether the parent system also offered a Medicare Advantage plan), and geographic scope (operating in one vs. two or more states). Enrollment and bed splits are at the median among provider-sponsored issuers. Subgroup characteristics were measured using CMS MLR data and the AHRQ Compendium of U.S. Health Systems. All models adjusted for plan type shares, metal tier shares, log member-years, and state and year fixed effects. Standard errors were clustered by issuer. The dashed line marks a difference of zero.

Supplement Table 1. Association Between Medical Loss Ratios and Overall Quality Ratings, Overall and by Issuer Type

	Base Model	With Interaction
Medical loss ratio, per 10 pp	0.149***	0.167***
	(0.030)	(0.031)
Provider-sponsored issuer	0.514***	0.550***
	(0.131)	(0.134)
Provider-sponsored x medical loss ratio		-0.071
		(0.061)
Observations	401	401
R-squared	0.553	0.556

Standard errors in parentheses
*P < .10, ** P < .05, *** P < .01.

Notes: Unit of observation is issuer-state-year from 2020 through 2023. The outcome is CMS Quality Rating System overall star rating on a 1- to 5-star scale. Medical loss ratio is centered at 80% and scaled per 10 percentage points. Column 2 includes an interaction between medical loss ratio and provider-sponsored issuer status; the interaction tests whether the MLR-quality association differs by issuer type. All models adjust for plan type shares, metal tier shares, log member-years, state fixed effects, and year fixed effects. Standard errors are clustered by issuer and shown in parentheses. MLR indicates medical loss ratio.

Supplement Figure 2. Association Between Provider-Sponsored Issuer Status and Medical Loss Ratios and Quality Improvement Expenses Across Robustness Samples

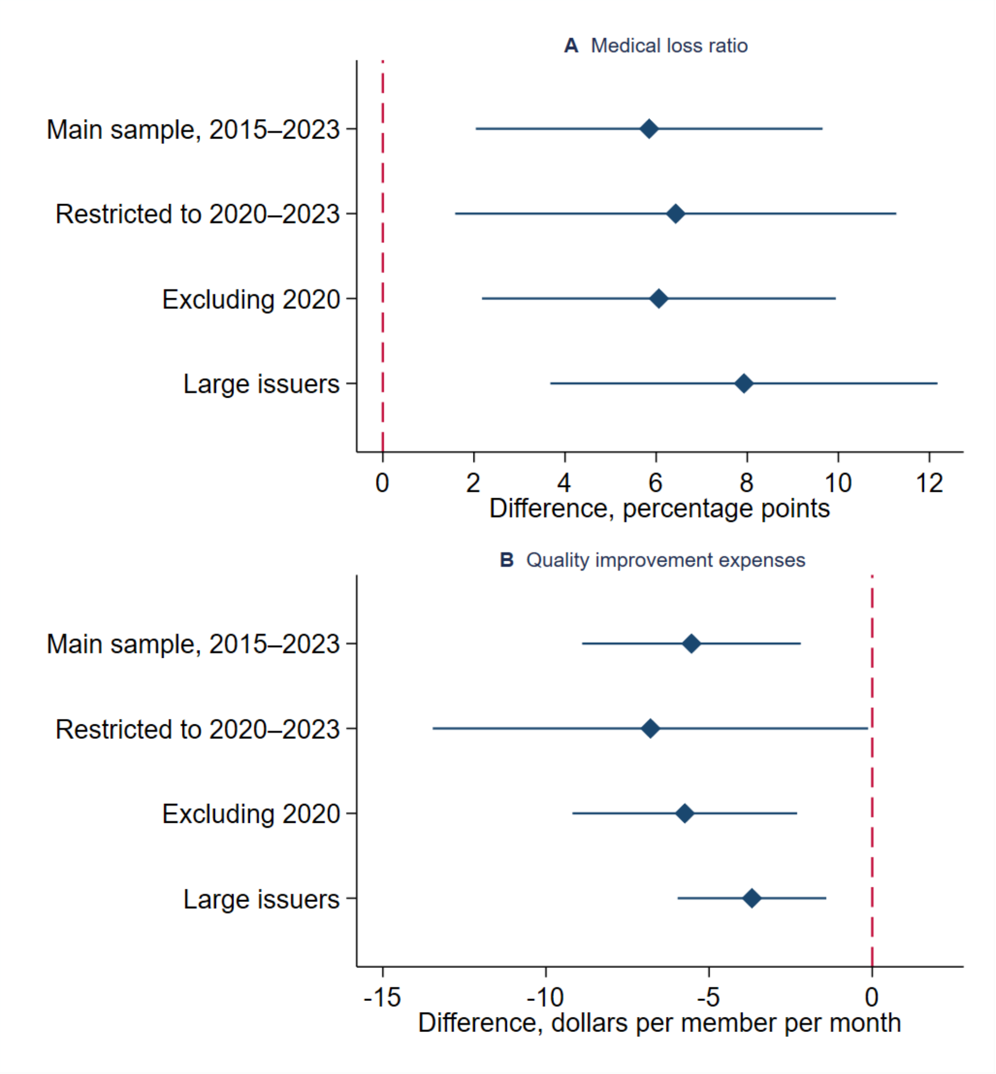

Notes: Each point represents the adjusted difference between provider-sponsored and traditional issuers, with 95% confidence intervals, estimated from a separate linear regression. Panel A reports medical loss ratios in percentage points and Panel B reports quality improvement expenses in dollars per member per month. Within each panel, estimates are shown for the main sample from 2015 through 2023, the sample restricted to 2020 through 2023, the sample excluding 2020, and issuers with at least 10,000 member-years. All models adjusted for plan type shares, metal tier shares, log member-years, and state and year fixed effects. Standard errors were clustered by issuer. The dashed line marks a difference of zero.

Supplement Figure 3. Association Between Provider-Sponsored Issuer Status and Reporting a Medical Loss Ratio below 0.80

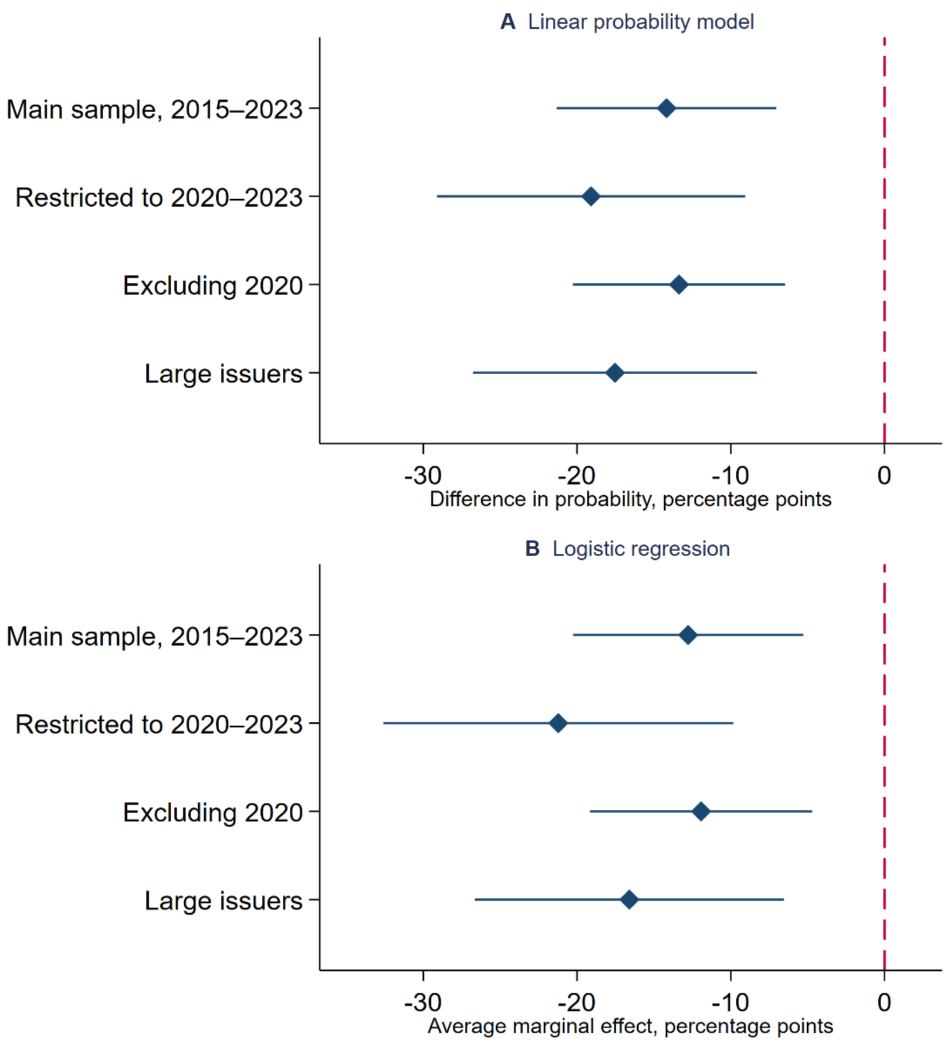

Notes: Each point represents the adjusted difference between provider-sponsored and traditional issuers in the probability of reporting a medical loss ratio below 0.80, the Affordable Care Act rebate threshold, with 95% CIs. Estimates are reported in percentage points. Panel A reports coefficients from linear probability models; Panel B reports average marginal effects from logistic regression models. Within each panel, estimates are shown for the main sample from 2015 through 2023, the sample restricted to 2020 through 2023, the sample excluding 2020, and issuers with at least 10,000 member-years. All models adjust for plan type shares, metal tier shares, log member-years, state fixed effects, and year fixed effects. Standard errors are clustered by issuer. The dashed line marks a difference of zero. MLR indicates medical loss ratio.
